# Role of Educational Level in Kidney Transplant Outcomes

**DOI:** 10.3390/biomedicines13040916

**Published:** 2025-04-09

**Authors:** Francesco Leonforte, Pierfrancesco Veroux, Antonio Mistretta, Alessia Giaquinta, Martina Maria Giambra, Domenico Zerbo, Giuseppe Roscitano, Concetta De Pasquale, Maria Luisa Pistorio, Antonio D’Anna, Carmelo Cusmano, Roberta Granata, Giordana Riccioli, Marianna Scribano, Massimiliano Veroux

**Affiliations:** 1 Public Health Physician, University Hospital Polyclinic “G. Rodolico San Marco”,95124 Catania, Italy; leonfortefrancesco1@gmail.com (F.L.); antoniodanna5@gmail.com (A.D.); cusmanocarmelo@gmail.com (C.C.); 2 Department of Medical and Surgical Sciences and Advanced Technologies, University of Catania, 95124 Catania, Italy; pveroux@unict.it (P.V.); anmist@unict.it (A.M.); 3 Vascular Surgery and Organ Transplant Unit, University Hospital of Catania, 95121 Catania, Italy; alessiagiaquinta@gmail.com (A.G.); giambramartina@gmail.com (M.M.G.); domenicozerbo@libero.it (D.Z.); giuseppe.roscitano@virgilio.it (G.R.); depasqua@unict.it (C.D.P.); m.luisapistorio@unict.it (M.L.P.); 4 General Surgery Unit, Azienda Policlinico San Marco, University of Catania, 95124 Catania, Italy; robertagranata@live.it (R.G.); giordanariccioli@me.com (G.R.); scribanomarianna@gmail.com (M.S.)

**Keywords:** kidney transplantation, living donor, deceased donor, graft survival, patient survival, employment, work, socioeconomic conditions, educational status, income, quality of life

## Abstract

**Background**: Kidney transplantation outcomes are correlated to many factors, including the socioeconomics conditions and the educational level. **Methods**: We evaluated the role of educational level on patient and graft survival in a population of 456 kidney transplant recipients. Patients were divided into two groups on the basis of their pre-transplant educational status: patients with primary education (elementary or middle school, as low education) were compared with patients with a secondary school education (high school or a university degree, as high education). **Results**: Among the 456 patients considered for this analysis, 161 patients had a low educational status, and 295 had a high educational status. Patients with a low educational status were more rarely employed (66.1% vs. 32.5%, *p* < 0.001), with a high rate of retired patients compared to high-educational status patients (35.6% vs. 10.6%, *p* < 0.001). Although the educational status did not influence the graft function, the 1-year (88.1% vs. 96.2%, *p* = 0.0008), 5-year (77.6% vs. 88.8%, *p* = 0.001), and 10-year (62.1% vs. 75%, *p* = 0.003) graft survival rates were significantly lower in patients with low educational status compared with high-educational status patients, respectively. Patient survival at 1-year (94.4% vs. 97.6%, *p* = 0.073), 5-year (85% vs. 92.5%, *p* = 0.011), and 10-year (75.7% vs. 83.4%, *p* = 0.042) follow-up was significantly lower in patients with low educational status. **Conclusions**: Low socioeconomic conditions and educational level had a negative impact on kidney transplant outcomes. Improving access to education and providing targeted educational support and health literacy could enhance treatment adherence and reduce disparities in transplant outcomes.

## 1. Introduction

Kidney transplantation (KT) is the best available treatment for patients with end-stage renal disease (ESRD); however, despite generally low long-term mortality risk after transplantation, recipients often face the challenge of allograft failure [1].

Health literacy, defined as the ability to acquire and comprehend health information to make suitable decisions, plays a significant role in healthcare utilization and patient outcomes. Poor health literacy is linked to worse blood pressure control, higher hospital admissions, lower rates of preventive screenings like mammography and flu vaccination, and increased healthcare costs [2,3]. However, there is limited research on health literacy in the ESRD population scheduled for KT. Transplant candidates constitute a heterogeneous group, including patients with a long-term dialysis treatment together with patients undergoing a preemptive transplantation. Limited health literacy is likely common among KT candidates, potentially affecting their success in becoming listed and surviving on the KT waiting list: up to 12.9% of KT candidates with less than a college education and 4.8% of those with a college education experience limited health literacy [4].

This limited health literacy was independently associated with reduced chances of listing for transplantation and increased mortality once listed. The highest rates of limited health literacy are found among those who are frail, cognitively impaired, or have less than a college education. Screening for health literacy during KT evaluations could be crucial in addressing disparities in transplant access and waitlist outcomes [4,5,6]. Primary care physicians, nephrologists, and dialysis providers might help by providing adequate guidance, coordinating medical clearance steps for KT, and offering additional support through transplant patient navigators, as well as increasing awareness of waitlist status [7,8,9]. Although improving health literacy is challenging among KT candidates, tailored pre- and post-KT programs may be helpful to increase the outcomes of those with limited health literacy [4].

The quality of life in ESRD patients could be significantly impaired by the dialytic treatment and dietary restrictions, particularly for those with inadequate health literacy [10]. Limited health literacy may affect up to 25% of ESRD patients and is variably linked to negative clinical outcomes and to a limited access to transplantation [10,11,12]. In contrast, only 14% of transplant recipients experience limited literacy, suggesting a selection process favoring those with higher literacy [9,11,12]. Interestingly, among transplant recipients, young women are at a higher risk of graft failure than young men, while older women face similar or lower risks compared to older men [13]. This could be due to earlier cognitive development in females, enhancing their capacity for self-care, or a stronger social desirability tendency that affects adherence [14,15].

Patients with low health literacy often have low socioeconomic status (SES), which correlates with poorer ESRD comprehension and health outcomes [12].

SES and its factors, such as education level, income, and insurance coverage, significantly impact patient prognosis. Higher SES is linked to better graft and patient survival, as well as improved access to healthcare resources, and many studies showed that higher education levels correlate with better health literacy and medication adherence, improving transplant outcomes [16,17,18,19]. For instance, lower education is associated with increased graft failure risk in Black patients, whereas White patients with lower education face a lower mortality risk [20]. Ghods et al. [21] suggest that lower education may lead to poor transplant outcomes through reduced adherence to immunosuppressive regimens, whereas higher education levels are associated with better healthcare access and sustained use of immunosuppressants. Furthermore, recipients with higher education typically receive more social support, which positively affects transplant success. Consistent findings show better transplant outcomes in recipients with higher education, reinforcing the link between education level and successful transplant outcomes [22,23,24].

Overall, these studies underscore the importance of educational attainment as a determinant of health outcomes and quality of life in kidney transplant recipients. Understanding these relationships can inform strategies to optimize patient care and outcomes in the field of renal transplantation. In this study, we investigated the impact of educational level on post-transplant outcomes, with a special focus on graft and patient survival.

## 2. Materials and Methods

In this study, we retrospectively evaluated the post-transplant outcomes of a cohort of kidney transplant recipients on the basis of their pre-transplant educational status: patients with primary education (elementary or middle school, as low education) were compared with patients with a secondary school education (high school or a university degree, as high education). Donor selection, kidney transplant procedures, and immunosuppressive therapy have been previously described in detail [25,26]. Delayed graft function was defined as the need for at least one dialysis session within one week after transplantation [26]. Primary kidney nonfunction was defined as the complete lack of functionality, so that the recipient never discontinued dialysis sessions after transplantation.

This study was approved by the Local Ethics Committee of the Azienda Policlinico San Marco of the University of Catania, and the patients provided written informed consent to undergo renal transplantation. A total of 456 consecutive patients who underwent a kidney transplantation from a deceased donor between January 2000 and December 2012 met the inclusion criteria and were included in this study.

### Statistical Analysis

This study included all recipients of deceased donor kidneys recruited over a 12-year period. Data were retrieved from a password-protected institutional database, where patients were de-identified. The aim of this study was to evaluate the impact of education level on both post-transplant survival and graft survival. The variable “education level” was dichotomized to increase statistical power by dividing the transplanted patients into two categories (0: elementary or middle school; 1: high school or university degree). Characteristics at the time of transplant of patients with low educational levels were compared with patients with high educational levels with the use of Fisher’s exact test, chi-square test, and Student’s *t*-test. Predictive factors of worse graft and patient survival with a *p*-value < 0.5 in univariate analysis were considered for the multivariate model using a downward stepwise binary logistic regression analysis. Data are expressed as mean ± standard deviation (SD). To compare parametric variables, either Pearson chi-square test or Fisher’s exact test was used. To compare nonparametric variables, either Student’s *t*-test or Mann–Whitney U test was used. The difference between the two means was calculated using the Wilson test. Odds ratios (ORs) were reported with a 95% confidence interval (95% CI) and *p*-values. The level of statistical significance was determined at *p* < 0.05. The primary analysis was a comparison of graft and patient survival rates with the Kaplan–Meier method. Since these were transplant patients, we assumed that the risks of graft loss and patient death would have decreased as time passed. We checked the fit of the Weibull model, assuming that the graph for the Weibull model with log(−log(S-estimate)) vs. log(_t) should resemble a straight line. Since the graph according to the Weibull model did not deviate much from a straight line, we performed the regression using the Weibull model. The statistical analyses were performed with Stata 18.

## 3. Results

A total of 456 kidney transplant recipients were collected in the study period.

The mean age was 47.4 ± 16.4 years, with a prevalence of male recipients (286 patients, 62.7%) and a mean BMI of 26.6 ± 9.4 kg/m^2^. The most frequent cause of end-stage renal disease was autosomal dominant polycystic kidney disease (91 patients, 19.9%), followed by glomerulonephritis (46 patients, 10.6%) and diabetes (22 patients, 4.8%), while the cause of ESRD was unknown in 162 (35.5%) of patients. Most patients (433 patients, 94.9%) were on hemodialysis at the time of transplantation, while seven patients (1.5%) received a pre-emptive kidney transplantation. Mean time on dialysis was 48 ± 23.2 months, while mean time on the waiting list was 19.2 ± 11.9 months. In the entire cohort, mean donor age was 51.4 ± 19.3 years, and a total of 23 donors (5.0%) had diabetes and 141 (30.9%) were hypertensive. A total of 161 patients had a low educational status, and 295 had a high educational status (Table 1).

Patients with low educational status were more frequently older and male and received a kidney from donors with a longer cold ischemia time and died from cerebral hemorrhage. Moreover, patients with a low educational level had a longer time on dialysis and experienced a higher rate of delayed graft function. This would suggest that patients with a low educational level and low socio-economic status had delayed access to the health system and to the transplant waiting list, and this would have an impact on transplant outcome. As expected, patients with a low educational status were more rarely employed (66.1% vs. 32.5%, *p* < 0.001), with a high rate of retired patients compared to high-educational status patients (35.6% vs. 10.6%, *p* < 0.001). There was no significant difference in graft functionality between the two groups, as demonstrated by serum creatinine levels at 1 year (1.41 ± 0.77 vs. 1.46 ± 0.80 mg/dL, *p* = 0.440), 5 years (1.66 ± 0.70 vs. 63 ± 10.4 mg/dL, *p* = 0.189), and 10 years (1.65 ± 0.90 vs. 1.53 ± 0.78 mg/dL, *p* = 0.367).

However, the educational status had an impact on graft survival and patient survival; 1-year (88.1% vs. 96.2%, *p* = 0.0008), 5-year (77.6% vs. 88.8%, *p* = 0.001), and 10-year (62.1% vs. 75%, *p* = 0.003) graft survivals were significantly lower in patients with a low educational status compared with high-educational status patients, respectively. Moreover, the Weibull model regression demonstrated that the organ failure event occurred 1.46 times faster in patients with low educational status. The low educational status had an impact also on the patient survival; 1-year (94.4% vs. 97.6%, *p* = 0.073), 5-year (85% vs. 92.5%, *p* = 0.011), and 10-year (75.7% vs. 83.4%, *p* = 0.042) patient survival rates were significantly lower in patients with low educational status.

**Figure 1:**
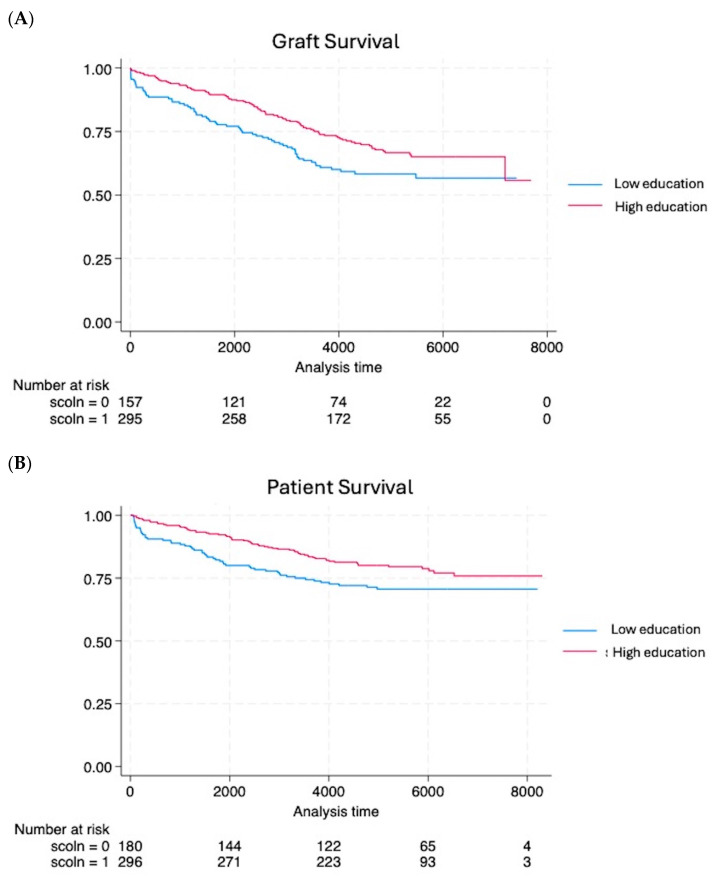
The Kaplan–Meier analysis demonstrated worse graft (**A**) and patient (**B**) survival in patients with low educational levels compared to patients with high educational levels.

The Kaplan–Meier analysis confirmed these findings: graft survival (Figure 1) was significantly higher in patients with a high education status (*p* = 0.018, OR = 0.68, 95% CI = 0.497–0.935), and also patient survival was significantly better in patients with a high educational level (*p* = 0.041, OR = 0.68, 95% CI = 0.472–0.984).

## 4. Discussion

Renal replacement therapy, including kidney transplantation, is a critical medical intervention for patients with ESRD, significantly impacting outcomes, costs, and quality of life improvements [6]. This study demonstrated a significant correlation between the education level of kidney transplantation recipients and post-transplant outcomes. Using a database of 456 patients, we analyzed demographic and clinical variables that could correlate with post-transplant graft and patient survival and education level. Kidney transplant recipients with a higher educational level had better survival than patients with a lower education level; the Weibull model fitting confirmed these differences, with the event of death occurring approximately 1.5 times faster in patients with a lower education level than those with a higher education level. Similar findings were found in graft survival: kidney transplant recipients with a higher education level had better graft survival compared with recipients with a low educational level. Again, the Weibull regression indicated that graft failure occurred approximately 1.46 times faster in low-education patients compared to patients with high education levels.

The educational level may influence the patients’ socioeconomic status and their ability to work, thereby influencing the quality of life; however, hemodialysis patients or kidney transplant recipients experience many difficulties in maintaining employment. A recent study [27] demonstrated that employment rates in patients with ESRD are very low and decline long before initiation of dialysis or kidney transplantation. Notably, the employment rate had only a small increase after kidney transplantation. Indeed, a recent systematic review [28] demonstrated that only 26% of patients undergoing dialysis were employed and that dialysis patients with diabetes and associated co-morbidities, such as peripheral vascular disease, heart failure, and cognitive decline, had the lowest chance of being employed. Kidney transplantation could help, in principle, to remove the barrier that prevents one from working effectively; a recent study [29] demonstrated that transplanted patients had an employment rate of 56%, and this was strictly correlated with higher education level, preemptive transplantation, and receiving a living donor transplant [29,30]. This was confirmed in this study, where 66% of patients with high educational status were employed after transplantation in contrast with the 32.5% of patients with a low educational status (*p* < 0.001). Moreover, patients with a low educational status retired more frequently compared with those with a high educational status. However, fatigue together with memory problems, restlessness, and anxiety could constitute an obstacle to returning to work for transplant patients, even in patients with a high educational level [29]. Moreover, transplanted patients worked shorter hours and were more likely to have jobs that are mentally rather than physically demanding [29].

A higher educational level is correlated with better access to healthcare, better treatment adherence, and better post-transplant outcomes [23,24]. This could be due to several factors: patients with a higher education level may have a better understanding of the importance of adhering to the post-transplant therapeutic regimen, including taking immunosuppressive drugs regularly and attending follow-up visits; furthermore, a higher level of education may be correlated with a greater ability to navigate the healthcare system and access quality medical resources.

Although the Italian healthcare system is designed to ensure equity in access to dialysis and transplants, socioeconomic disparities continue to influence clinical outcomes and therapeutic adherence. Patients with a lower level of education are often less informed and less able to manage the complex therapeutic regimens required after transplantation, which could lead to a higher incidence of complications and reduced graft survival [18]. This was confirmed in our study: kidney transplant recipients with a low educational level had a longer time on dialysis and delayed access to the transplant health system, and these factors could negatively influence the post-transplant outcomes. Moreover, patients with a low educational status were more rarely employed, with a high rate of retired patients compared to high-educational status patients, and this could influence the perceived better quality of life associated with kidney transplantation [10,31].

Interestingly, when the socioeconomic status improves in the post-transplant period, post-transplant outcomes are positively influenced. In contrast, patients whose economic status did not improve 3 years after kidney transplantation showed a higher risk of death than those whose status improved [32].

Lower educational status has been associated with lower health literacy, which can affect a patient’s ability to manage their health effectively and adhere to post-transplantation treatment protocols [20]. In contrast, higher educational status is often linked to better health outcomes due to better access to healthcare resources, increased social support, and a greater ability to comply with medical advice [21,22]. It is important to highlight some limitations of our study. The measurement of education level might be subject to misclassification bias, and the data collected might not be complete due to patients’ reluctance to share certain information. Moreover, the relatively small sample size and the lack of data on some potential confounding factors might affect the generalizability of the results. Although our study showed an association between education level and post-transplant outcomes, it cannot establish a direct causal relationship. Future studies with larger samples and longitudinal study designs are needed to further explore these findings and better understand the mechanisms through which education may influence clinical outcomes. In conclusion, our study underscored the importance of considering socioeconomic factors, such as education level, in managing patients who have undergone kidney transplantation. Improving access to education and providing targeted educational support and health literacy could enhance treatment adherence and reduce disparities in transplant outcomes. Moreover, a correct and early education about kidney transplantation could help design a retraining option, resulting in the adaptation of the working conditions to the patient’s needs. This approach could lead to significant improvements in the quality of life and survival of transplanted patients, contributing to a more equitable and inclusive healthcare system.

## Figures and Tables

**Table 1:** Clinical characteristics and comparison between the recipients with low educational status (n = 161) and those with high educational status (n = 295).

Groups and their Characteristics	Low Educational Status (n = 161)	High Educational Status (n = 295)	*p*-Value
**Donor**			
Age (year)	54.1 ± 16	48.7 ± 19.3	0.005
Male sex (%)	78 (48.7)	108 (36.4)	0.013
Terminal serum creatinine (mg/dL)	1.06 ± 0.3	1.20 ± 0.7	0.061
Use of vasoactive amines (%)	155 (96.8)	275 (92.9)	0.832
Diabetes (%)	8 (5)	15 (5)	0.343
Cold ischemia time (h)	16.6 ± 5.1	15.2 ± 5.8	0.017
Cerebral hemorrhage/ischemia brain death (%)	122 (76.2)	185 (62.5)	0.006
Traumatic brain death (%)	31 (19.3)	95 (32.0)	0.003
Other causes of brain death	8 (5)	19 (6.4)	0.617
Stay in the ICU	5 ± 3.4	5.1 ± 3.9	0.824
**Recipient**			
Age (year)	52.08 ± 10.9	46.6 ± 12.4	<0.001
Male sex (%)	94 (58.3)	194 (65.7)	0.138
Pre-transplant panel reactive antibody (%)	24 ± 8.9	19 ± 8.5	0.774
BMI (mean, kg/m^2^)	27.4	26.2	0.645
Time on dialysis (mo)	61.9 ± 60	41.3 ± 41	<0.001
Time on waiting list (mo)	20.6 ± 28	18.9 ± 25	0.502
Hemodialysis (%)	159 (98.7)	279 (94.5)	0.885
Peritoneal dialysis (%)	0 (0)	10 (3.3)	0.433
Pre-emptive	1 (1.3)	7 (2.3)	0.173
Dual transplant (%)	16 (10)	28 (9.4)	0.883
Delayed graft function (%)	82 (51.2)	80 (27.1)	<0.001
Discontinuation of dialysis (dy)	5.43 ± 3.1	3.3 ± 7.1	0.002
Primary non-function (%)	8 (5)	6(2.5)	0.733
Acute rejection	16 (6.7)	25 (10.5)	0.122
**Immunosuppression**			
Induction (basiliximab)	45 (28.1)	78 (23.1)	0.753
Induction (thymoglobuline)	22 (13.7)	44 (10)	0.883
Tacrolimus	110 (68.5)	204 (68.9)	0.214
MMF	140 (87.5)	250 (77.7)	0.301
Sirolimus	24 (15)	48 (10.9)	0.112
Cyclosporine	25 (15.6)	59 (23.1)	0.108
Everolimus	12 (7.5)	21 (7.9)	0.323
**Hospital stay**	9.8 ± 3.4	10.5 ± 5.1	0.242
**Postoperative death (30-day)**	3 (1.8)	4 (1.3)	0.738
**Employed (%)**	52 (32.5)	195 (66.1)	<0.001
**Part-time employed (%)**	2 (1.25)	11 (3.7)	0.128
**Housewife (%)**	24 (15)	28 (9.4)	0.080
**Retired (%)**	57 (35.6)	31 (10.5)	<0.001
**Unemployed (%)**	12 (7.5)	25 (8.4)	0.702
**1-year serum creatinine (mg/dL)**	1.41 ± 0.77	1.46 ± 0.80	0.490
**5-year serum creatinine (mg/dL)**	1.66 ± 0.70	1.54 ± 0.78	0.189
**10-year serum creatinine (mg/dL)**	1.65 ± 0.90	1.53 ± 0.78	0.367
**1-year eGlomerular Filtration Rate (mL/min per 1.73 m^2^)**	65 ± 12.4	68 ± 9.5	0.012
**5-year eGlomerular Filtration Rate (mL/min per 1.73 m^2^)**	61 ± 11.4	63 ± 10.4	0.048
**10-year eGlomerular Filtration Rate (mL/min per 1.73 m^2^)**	58 ± 12.3	59 ± 11.8	0.633
**1-year patient survival**	94.4%	97.6%	0.073
**5-year patient survival**	85%	92.5%	0.011
**10-year patient survival**	75.7%	83.4%	0.042
**1-year graft survival**	88.1%	96.2%	0.0008
**5-year graft survival**	77.6%	88.8%	0.001
**10-year graft survival**	62.1%	75%	0.003

## Data Availability

It is possible for de-identified data to be made available upon reasonable request.
